# Exploring the Electron Transfer Pathway in the Oxidation of Avermectin by CYP107Z13 in *Streptomyces ahygroscopicus* ZB01

**DOI:** 10.1371/journal.pone.0098916

**Published:** 2014-06-06

**Authors:** Mei Li, Yujie Zhang, Lin Zhang, Xiaoyan Yang, Xiliang Jiang

**Affiliations:** State Key Laboratory for Biology of Plant Diseases and Insect Pests, Institute of Plant Protection, Chinese Academy of Agricultural Sciences, Beijing, China; Center for Nanosciences and Nanotechnology, Mexico

## Abstract

*Streptomyces ahygroscopicus* ZB01 can effectively oxidize 4″-OH of avermectin to form 4″-oxo-avermectin. CYP107Z13 is responsible for this site-specific oxidation in ZB01. In the present study, we explored the electron transfer pathway in oxidation of avermectin by CYP107Z13 in ZB01. A putative [3Fe-4S] ferredoxin gene *fd*68 and two possible NADH-dependent ferredoxin reductase genes *fdr*18 and *fdr*28 were cloned from the genomic DNA of ZB01. *fd*68 gene disruption mutants showed no catalytic activity in oxidation of avermectin to form 4″-oxo-avermectin. To clarify whether FdR18 and FdR28 participate in the electron transfer during avermectin oxidation by CYP107Z13, two whole-cell biocatalytic systems were designed in *E. coli* BL21 (DE3), with one co-expressing CYP107Z13, Fd68 and FdR18 and the other co-expressing CYP107Z13, Fd68 and FdR28. Both of the two biocatalytic systems were found to be able to mediate the oxidation of avermectin to form 4″-oxo-avermectin. Thus, we propose an electron transfer pathway NADH→FdR18/FdR28→Fd68→CYP107Z13 for oxidation of avermectin to form 4″-oxo-avermectin in ZB01.

## Introduction

**Figure 1:**
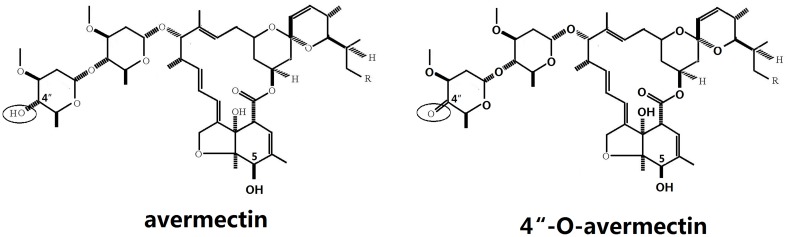
Structures of the avermectin and product 4″-O-avermectin.

*Streptomyces* spp. produces many important natural products, including many known antibiotics. Cytochrome P450 enzymes (CYP450s) are involved in these biosynthetic and biotransformation reactions [1], [2]. P450s are heme-dependent monooxygenases that catalyze the insertion of oxygen atoms from atmospheric oxygen molecules into carbon–hydrogen bonds within a diverse range of organic compounds [3]. Emamectin benzoate is a derivate of avermectin, a potent semisynthetic insecticide used to control many agriculturally important pests. Oxidation of 4″-OH into 4″-oxo of avermectin is one key reaction step in the synthesis of emamectin benzoate from avermectin [4]. Direct regiospecific chemical oxidation of the 4″-OH group in avermectin to form 4″-oxo-avermectin is precluded by the high reactivity of the 5-OH group in the molecule, necessitating a protection–deprotection strategy (Fig. 1). Avoiding these additional steps would greatly reduce the complexity of the production process along with the final cost of emamectin benzoate. CYP107Zs from *Streptomyces* were reported to have the capability to oxidize 4″-OH into 4″-oxo of avermectin regioselectively [5].

Many CYP450s from bacterial were found to be class I type electron transfer systems. Including CYP153 family from gram-positive alkane-degrading bacteria [6], [7], CYP199A4 from *Rhodopseudomonas palustris* HaA2 [8], CYP105 family, CYP107 family and other CYPs from *Streptomyces* [3], [5], [9], [10]. Classical class I type electron transfer system, consists of an FAD-containing ferredoxin reductase (FdR), an iron–sulfur protein ferredoxin (Fd), and a cytochrome P450 (P450) [11]. Electrons are delivered from the reduced pyridine nucleotide coenzymes NAD(P)H to P450 via Fd and FdR [12]. and the Fd are usually [2Fe-2S] type, although there are also reports on the use of ferredoxins of other cluster types. In *S. griseus*, both a [3Fe-4S] ferredoxin and a 7 Fe ferredoxin, that contains a [3Fe-4S] as well as a [4Fe-4S] cluster, were shown to deliver electrons to CYP105D1 (P450soy) [3]. In *Bacillus subtilis*, a [4Fe-4S] cluster ferredoxin was suggested as a potential redox partner of CYP107H (P450BioI) [13]. Only a few Fds and FdRs from bacterial P450 systems have been purified and characterized because of their instability and relatively low expression levels [14]–[16].

In our previous study, we screened an *S. ahygroscopius* strain ZB01 which can oxidize 4″-OH of avermectin to form 4″-oxo- avermectin with greater efficiency than those of reported functional *Strept*omyces [15], [17]. CYP107Z13 was found to be responsible for this site-specific oxidation in ZB01 [18]. In this study, we explored the electron transfer process in the oxidation of avermectin by CYP107Z13 in ZB01. A ferredoxin gene *fd*68 and two ferredoxin reductase genes *fdr*18 and *fdr*28 were cloned from ZB01, and we found that there exist an electron transfer pathway NADH→FdR18/FdR28→Fd68→CYP107Z13 in ZB01 for oxidation of avermectin to form 4″-oxo-avermectin.

## Materials and Methods

### Bacterial Strains and Plasmids

The microorganisms and plasmids used in this study are listed in Table 1. *S. ahygroscopicus* ZB01 (CGMCC No. 2804) was isolated and maintained in our laboratory, and was grown in liquid YEME medium or on YMS agar [19]. The protoplast regeneration medium was R2YE [19]. *E. coli* DH5α (Trans, Beijing) was used for bacterial transformation and plasmid propagation. *E. coli* BL21 (DE3) (Trans, Beijing) was used for recombinant protein expression and whole-cell biocatalytic systems. For the plasmid-containing cultures, 100 µg ml^−1^ ampicillin and/or 50 µg ml^−1^ kanamycin for *E. coli* strains or G418 (10 µg ml^−1^ for *E. coli* strains and 5 µg ml^−1^ for *Streptomyces* strains) instead of apramycin were added.

**Table 1:** Microoganisms and plasmids used in this study.

Strain or plasmid		Source
*E. coli* DH5α	Routine cloning host	Beijing TransGen Biotech Co. Ltd.
*E. coli* BL21 (DE3)	T7 system expression host	Beijing TransGen Biotech Co. Ltd.
*E. coli z13*	E. coli BL21 (DE3) containing pRSET-*z13*	This study
*E. coli fdr*18	*E. coli* BL21 (DE3) containing pRSET-*fdr*18	This study
*E. coli fdr*28	*E. coli* BL21 (DE3) containing pRSET-*fdr*28	This study
*E. coli*-*zfr*18	*E. coli* BL21 (DE3) containing pRSET-z13 and pDuet-*fd*-*fdr*18	This study
*E. coli*-*zfr*28	*E. coli* BL21 (DE3) containing pRSET-z13 and pDuet-*fd*-*fdr*28	This study
*S. ahygroscopicus* ZB01	CGMCC 2804, cyp107z13, *fd*68, *fdr*18 and *fdr*28 producer	This lab
ZBΔ*fd*68-3	*fd* 68 disruption mutant of *S. ahygroscopicus* ZB01	This study
ZBΔ*fd*68-6	*fd*68 disruption mutant of *S. ahygroscopicus* ZB01	This study
pMD19-T Easy	TA cloning vector, Amp^R^	This study
pKC1139	*Streptomyces*-*E. coli* conjugative shuttle vector, Am^r^	Bierman et al. (1992)
pKC1139::*fd*68	172 bp fragment of *fd*68 into *Hind* III- and *EcoR* I-cut pKC1139	This study
pRSET-b	Expression vector in *E. coli*, Amp^R^	Novagen,
pRSET-*fdr*18	pRSET-b carrying *fdr*18	This study
pRSET-*fdr*28	pRSET-b carrying *fdr*28	This study
pRSET-z13	pRSET-b carrying *cyp107z13*	This study
pRSFDuet-1	Vector for co-expressing two proteins in *E. coli*, Km^R^	Novagen
pDuet-*fd*-*fre*18	pRSFDuet-1 carrying *fd*68 and *fdr*18	This study
pDuet-*fd*-*fre*28	pRSFDuet-1 carrying *fd*68 and *fdr*28	This study

### Cloning of Ferredoxin and Ferredoxin Reductase Genes

Genomic DNA of *S. ahygroscopius* ZB01 was prepared according to Kieser et al. (2000) and used as the template for PCR reaction. Primers used for PCR are listed in Table 2. According to the conserved region of the flanking sequence of Fd genes from *Streptomyces* in NCBI, a pair of primers Fd1 and Rd1 were designed for cloning Fd gene in ZB01. PCR amplification was performed using 1 µM primers and LA Taq polymerase with GC buffer (TaKaRa, Japan). The PCR program was as follows: denaturation at 94°C for 4 min; followed by 30 cycles of 94°C for 1 min, 62°C for 1 min, and 72°C for 2 min; and a final extension of 72°C for 10 min. For cloning FdR genes from ZB01, Primer pairs F1/R1 and F2/R2 were designed based on the known FdR genes in NCBI to amplify the full-length and partial FdR gene fragment respectively in ZB01 in combination with the La Taq DNA polymerase in GCI buffer (TaKaRa, Japan). The PCR program used was as follows: denaturation at 94°C for 5 min; 30 to 32 cycles of 94°C for 1 min, 63°C for 1 min, and 72°C for 1.5 min; and a final extension at 72°C for 10 min. The resulting 1.3-kb PCR product (*fdr*18 gene) using F1/R1 and the 0.6-kb PCR product (partial sequence of *fdr*28 gene) using F2/R2 were cloned into the pMD19-T vector system. The correct clones were confirmed by sequencing. The full-length *fdr*28 gene sequence was cloned using a genome walking kit (TaKaRa, Japan). Three rounds of thermometric asymmetric nested PCR were performed using arbitrary primers (AP1–AP4) and specific primers (spF1-3 and spR1-3 were used to amplify the 3′ and 5′ flanking regions of the 0.6-kb known sequence, respectively). All the PCR products were cloned into the pMD18-T vector system and the correct clones were confirmed by sequencing. Plasmid manipulation, transformation of *E. coli*, restriction digestion, DNA fragment isolation and cloning techniques were performed according to standard procedure [19], [20] and the manufacturer’s instructions.

**Table 2:** Primers used for PCR in this study.

Primer Name	Nucleotide sequences (5′–3′)	Enzyme site	Products
Fd1	GGATACCTCGCGGATCTGATGG	None	
Rd1	ACAGCGGCGGGATGAAGGAC	None	*fd*68
F1	GTGGTCGACGCACACCAGACG	None	
R1	CTACGGGAGCAGTGMSGYCAGC	None	*fdr1*8
F2	GAACACGCCGAAGCCCTCCA	None	
R2	CAGAACAACGGCAGGGTCAGG	None	0.6 kb *fdr*28 gene fragment
spF1	ACTCGGAGGCATCCGCAACA	None	
spF2	TGCCCGAACCCGATCTACGA	None	
spF3	GCCCATCAACCGCAGTGCCT	None	
spR1	CGTGCAGATGATGATGACGACCC	None	
spR2	TCGCCTCGGACCTCATGGACTA	None	
spR3	ACCGCTGCCCACTGCACTCA	None	full-length *fdr*28 gene
tF	TA*GAATTC* **ATG**GTCGACGCACACCAGACG	*EcoR* I	
tR	TT*AAGCTT* CTACGGGAGCAGTGACGTCAGCG	*Hind* III	*fdr*18
eF	TA*AGATCT* **ATG**GTTGTCGGAGCGTCACT	*Bgl* II	
eR	TT*AAGCTT* CTACCGGTGCTGGTACGCGGCCGT	*Hind* III	*fdr*28
Fd2	ATT*AAGCTT*GGCGTCTGTATCGGTTCCGGTC	*Hind* III	
Rd2	ATA*GAATTC*CTCCGTGACCTCGATGGCCTGTA	*EcoR* I	△*fd*68
AF1	GCTCATCGGTCAGCTTCTCAACCTTGG	None	
AR1	CACCTGTCCGCCAAGGCAAAGC	None	apramycin resistance gene
fF1	ATGCGGATCACCATCGACACC	None	
fR1	TCGGCGTCAGTCCTCCGTGA	None	*fd*68
z13F	GA*AGATCT*ATGACCGAACTAACGGACTCCCC	*Bgl* II	
z13R	CG*GAATTC*TCAGTTCAACCGCAGCGGCAG	*EcoR* I	*cyp107z13*
RfdF	GAAGATCTATGCGGATCACCATCGACACCG	*Bgl II*	
RfdR	GGGGTACCTCAGTCCTCCGTGACCTCGATGG	*Kpn I*	*fd68*
Rzre1F	AAAGAATTCATGGTCGACGCACACCAGACG	*EcoR I*	
Rzre1R	AAAAAGCTTCTACGGGAGCAGTGACGTCAGC	*Hind III*	*fdr18*
Rzre2F	TAGAATTCATGGTTGTCGGAGCGTCACT	*EcoR I*	
Rzre2R	TTAAGCTTCTACCGGTGCTGGTACGCGGCCGT	*Hind III*	*fdr28*

### Disruption of Ferredoxin Gene

Fd gene was inactivated by gene disruption via single-crossover recombination. A 172 bp fragment from *fd*68 named Δ*fd*68 was amplified by PCR with primers Fd2/Rd2 and genomic DNA of *S. ahygroscopicus* ZB01 as the template. The PCR product was then subcloned into the *Hind* III/*EcoR* I digested pKC1139 [21] to generate the gene disruption vector pKC1139:: *fd*68. pKC1139:: *fd*68 was propagated in *E. coli* DH5α and transformed into *S. ahygroscopicus* ZB01 protoplasts mediated by PEG [22]. The transformants were selected for G418 resistance, and were then induced by a high temperature at 39°C for 48 h to obtain *fd*68 disruption mutant strains. The disruption strains were cultivated on YMS medium [19] for more than 3 generations without G418 selection for obtaining stable resistant transformants. Apramycin resistance gene and *fd*68 gene were analyzed by PCR for confirmation that *fd*68 disruption mutants have the correct structure. The colony morphologies were observed with the naked eye. Sporulation was checked using optical microscope. The growth rates of strains were measured by analysis of mycelium biomasses [19]. Each experiment was repeated for three times.

### Expression, Purification and Characterization of Ferredoxin Reductases

For expression FdR proteins in *E. coli*, pRSET-*fdr*18 plasmid was constructed by inserting *fdr*18 gene into *EcoR* I and *Hind* III sites of pRSET-b. pRSET-*fdr*28 plasmid was constructed by inserting the *fdr*28 gene into *Bgl* II and *Hind* III sites of pRSETb. Primers tF/tR and eF/eR were used for amplifying *fdr*18 and *fdr*28 genes respectivly, where the native GTG start codons were changed into ATG (in bold) to facilitate the expression in *E. coli* (Table 2). *E. coli* BL21 (DE3) containing the expression constructs were grown in LB medium supplemented with 100 µg ml^–1^ ampicillin at 37°C until OD_600_ reached 0.6. Isopropyl-thio-β-d-galactopyranoside (IPTG) was used as the inducer and δ-aminolevulinic acid was used as the heme precursor at a final concentration of 0.5 mM. The strain was allowed to grow for 6 h at 28°C. The cells were used for extracting recombinant proteins. The recombinant proteins were purified through Ni-Sepharose 6 fast flow column (GE Healthcare) and eluted with elution buffer with 200 mM imidazole, and were further concentrated and stored at −20°C with 10% glycerol for further use. The samples were analyzed on 12.5% sodium dodecyl sulfate-polyacrylamide gel electrophoresis (SDS-PAGE) and the proteins were visualized with coomassie brilliant blue staining.

Optical spectra of the recombinant FdR18 and FdR28 were recorded on a MAPADA UV-6100S Bio spectrophotometer (Shanghai Metash). The activities of the proteins were tested spectrophotometrically by determining the electron transfer rates from FdRs to DCPIP with NADH or NADPH as reducing agents according to method of Kirsty [23].

### Co-expression of CYP107Z13, Fd and FdR in *E. Coli*

Two compatible plasmids, pRSET-b and pRSFDuet-1, were used for co-expressing cytochrome P450 gene cyp107z13, *fd*68 and *fdr*18/*fdr*28 in *E. coli* BL21(DE3). pRSET was used for expressing CYP107Z13, A 1.5 kb PCR product of *cyp107z13 *DNA fragment with z13F/z13R (table 2) as primers was cloned into the pRSET-b vector at *Bgl* II and *EcoR* I sites to produce pRSET-z13. pRSFDuet-1(Novagen) was used for co-expression of Fd and FdR in *E. coli* BL21(DE3). Full length *fd*68, *fdr*18 and *fdr*28 gene fragments were amplified by PCR from the genomic DNA of ZB01. The PCR primers RfdF/RfdR were used for amplifying *fd*68, Rzre1F/Rzre1R for *fdr*18, and Rzre2F/Rzre2R for *fdr*28. All the native GTG start codons were changed into ATG (in bold) (Table 2). *fd*68 DNA fragment was cloned into the *Bgl*II and *Kpn*I sites and *fdr*18 was cloned into the *EcoR*I and *Hind*III sites of pRSFDuet-1 to generate pDuet-*fd*-*fdr*18. pDuet-*fd*-*fdr*28 were constructed by insertion *fre*28 fragment instead of *fre*18 into pDuet-*fd*-*fdr*18. All cloning results were verified by DNA sequencing.

pRSET-*z13* and pDuet-*fd*-*fdr*18 were co-transformed into *E. coli* BL21(DE3) for co-expression of CYP107Z13, Fd68 and FdR18. pRSET-z13 and pDuet-*fd*-*fdr*28 were co-transformed for co-expression of CYP107Z13, Fd68 and FdR28 by method of Hanahan [24]. Dual screening of ampicillin (100 µg ml^−1^) and kanamycin (50 µg ml^−1^) were used to maintain stable expression. *fd*68, *cyp107z13*, *fre18* and *fre28* genes in the *E. coli* BL21 (DE3) transformants containing two expression constructs were verified by PCR. The expressed proteins of those transformants were analyzed on 12.5% SDS-PAGE, and the proteins were visualized with coomassie brilliant blue staining.

### Avermectin Catalytic Activity Detection

Spores of *S. ahygroscopicus* ZB01 and *fd*68 disruption mutant strains were grown in ISP-2 liquid medium at 30°C with shaking at 200 rpm for two days. 100 mg avermectin ml^−1^ in isopropanol was then added and the spores were cultured for another two days. Avermectin and their derivatives were extracted with methyl-*t*-butyl ether, collected and redissolved in acetonitrile, and were finally detected using HPLC [18].

For detection of the whole-cell biocatalytic activities of *E. coli* BL21(DE3), co-expressing CYP107Z13, Fd and FdR, the strains were cultured in 3 ml LB with 100 µg ml^−1^ ampicillin and 50 µg ml^−1^ kanamycin at 37°C for 8 h and then transferred into 30 ml fresh LB, supplemented with appropriate antibiotics, and cells were allowed to grow at 37°C for 2 h. And then 0.5 mM *a*-aminolevulinic acid (ALA), 0.5 mM IPTG and 100 µg ml^−1^ avermectin in isopropanol were added to the cultures. After further incubation for 6 h at 28°C, the cultures were processed for detecting avermectin and its derivatives using HPLC [18].

### Bioinformatic Analysis

Sequence similarity analysis and alignment were carried out using the BLASTX, DNAman (5.0), and CLUSTAL X programs. The amino acid sequence was predicted using the SWISSPORT database via ExPASy. The isoelectric points and molecular weights of the predicted proteins were calculated using a PROTPARAM tool and DNAman (5.0). The online tool SignalP 4.1 was used for signal peptide analysis.

### Nucleotide Sequence Accession Numbers

The nucleotide sequences of ferredoxin gene *fd*68, ferredoxin reductase genes *fdr*18 and *fdr*28 reported in this paper were deposited into the GenBank database under accession numbers **KC147630**, **KC147631** and **KC510106**, respectively.

## Results

### Cloning and Sequence Analysis of Ferredoxin Gene *fd*68, Ferredoxin Reductase Genes *fdr*18 and *fdr*28

**Figure 2:**
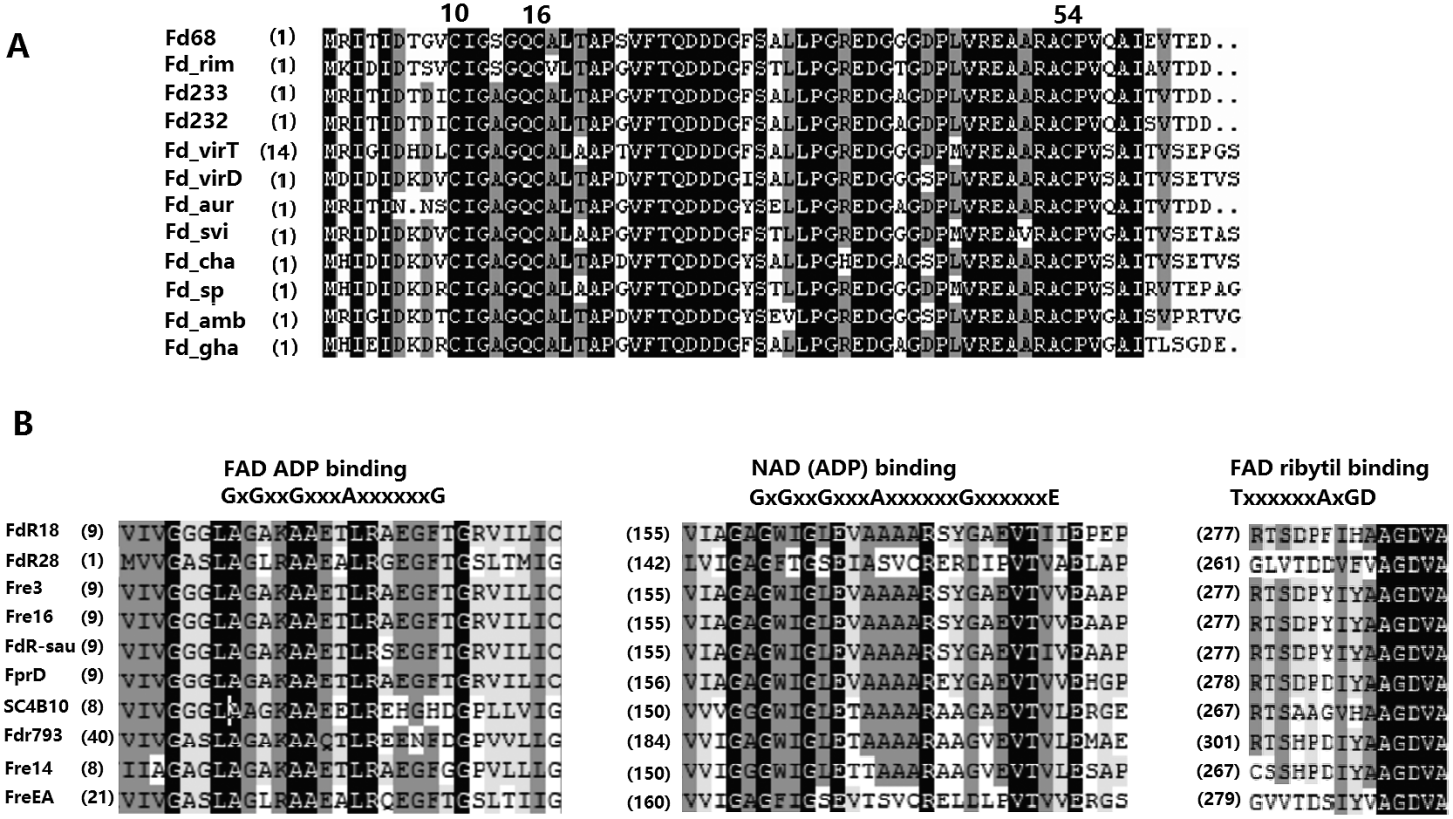
Sequence alignments of ferredoxins and ferredoxin reductases. (A) Alignment of the amino acid sequence of Fd68 from *S. ahygroscopicus* ZB01 with different 3Fe-4S-Fds. Fd_rim(**ZP_20965346**) is from *S. tubercidicus*;Fd233(**AY549200**) is from *S. tubercidicus* R-922; Fd232(**AY552101**) is from *S. tubercidicus* I-1529; Fd_virT(**WP_003995703**) is from *S. Viridochromogenes*; Fd_virD(**ZP_07308348**) is from *S. viridochromogenes* DSM 40736; Fd_aur(**ZP_10545293**) is from *S. auratus* AGR0001; Fd_svi(**ZP_06921672**) is from *S. sviceus* ATCC 29083; Fd_cha(**ZP_06921672**) is from *S. sviceus* ATCC 29083; Fd_sp(**BAG55293**) is from *Streptomyces* sp. A-1544; Fd_amb(**CAJ88533**) is from *S. ambofaciens* ATCC 23877; Fd_gha(**ZP_06574442**) is from *S. ghanaensis* ATCC 14672. (B) Multiple sequence alignment of conserved regions in FdRs. Consensus sequences for the FAD ADP-binding motifs, the NAD (ADP)-binding motifs, and the FAD ribytil-binding motifs (Asturis 1995) are shown above the alignment. FdR18, FdR28 are the FdRs described in this study. FprD(**NP_826852.1)** is from *S. avermitilis* MA-4680. Fre3(**AAT45306.1)**, Fre14(**AAT45308.1)**, Fre16(**AAT45309.1)**, FreEA(**AAT45279.1**) are from the genome of *S. tubercidicus* (Molnar, 2005). Fdr793(**AJ628764.1)** is of *S. peucetius* ATCC27952. SCF15(**CAB60462.1**) and SC4B10(**CAC04223.1**) are of *S. coelicolor* A3(2); FdR-sau(sau, **EJJ04871.1**) is of *S. auratus* AGR0001.

A 1810 bp DNA fragment was cloned from *S. ahygroscopicus* ZB01 genome by PCR with primers F1/R1, and an open reading frame of 195 bp within the fragment was obtained and named *fd*68. The GC content of *fd*68 was as high as 71.3%. The deduced Fd68 contains 63 amino acids with a molecular weight of 7.1 kDa and was a putative [3Fe-4S] Fd, with the conserved amino acid binding sites coordinated with [3Fe-4S] type iron sulfur cluster [25] at Cys10, 16 and 54 (Fig. 2A). Fd68 exibits 89.1% identity and 88.9% similarity to Fd232 of *S. tubercidicus* I-529 and Fd233 of *S. tubercidicus* R-922 [5].

FdR gene from ZB01 DNA was amplified by PCR using primers F1/R1 and was named *fdr*18. A 600 bp fragment was amplified using primers F2/R2, and the upstream and downstream flanking sequence regions were obtained by three rounds of nested PCR. The combined full-length FdR gene was named *fdr*28.

*fdr*18 contains 1263 bp and encodes a 420 amino acid protein with a molecular weight of 45.0 kDa. *fdr*28 contains 1344 bp and encodes a 447 amino acid protein with a molecular weight of 47.8 kDa. The pI of FdR18 was 5.28 and that of FdR28 was 5.57. FdR18 has the highest similarity (95%) to FdR from *S. rimosus* ATCC 10970, whereas FdR28 has the highest similarity (92%) to FdR of *S. violaceusniger* Tu 4113. The sequence similarity between FdR18 and FdR28 was only 29.81%. Both FdR18 and FdR28 contain the conserved putative flavin adenine dinucleotide (FAD) – and Nicotine ademine dinucleotide (NAD) – binding sites, and FAD ribytil-binding motifs (Fig. 2B), and neighter of them have signal peptide sequences, indicating that FdR18 and FdR28 were not extracellularly secreted proteins.

### Biological Characteristics of *fd*68 Disruption Mutants

**Figure 3:**
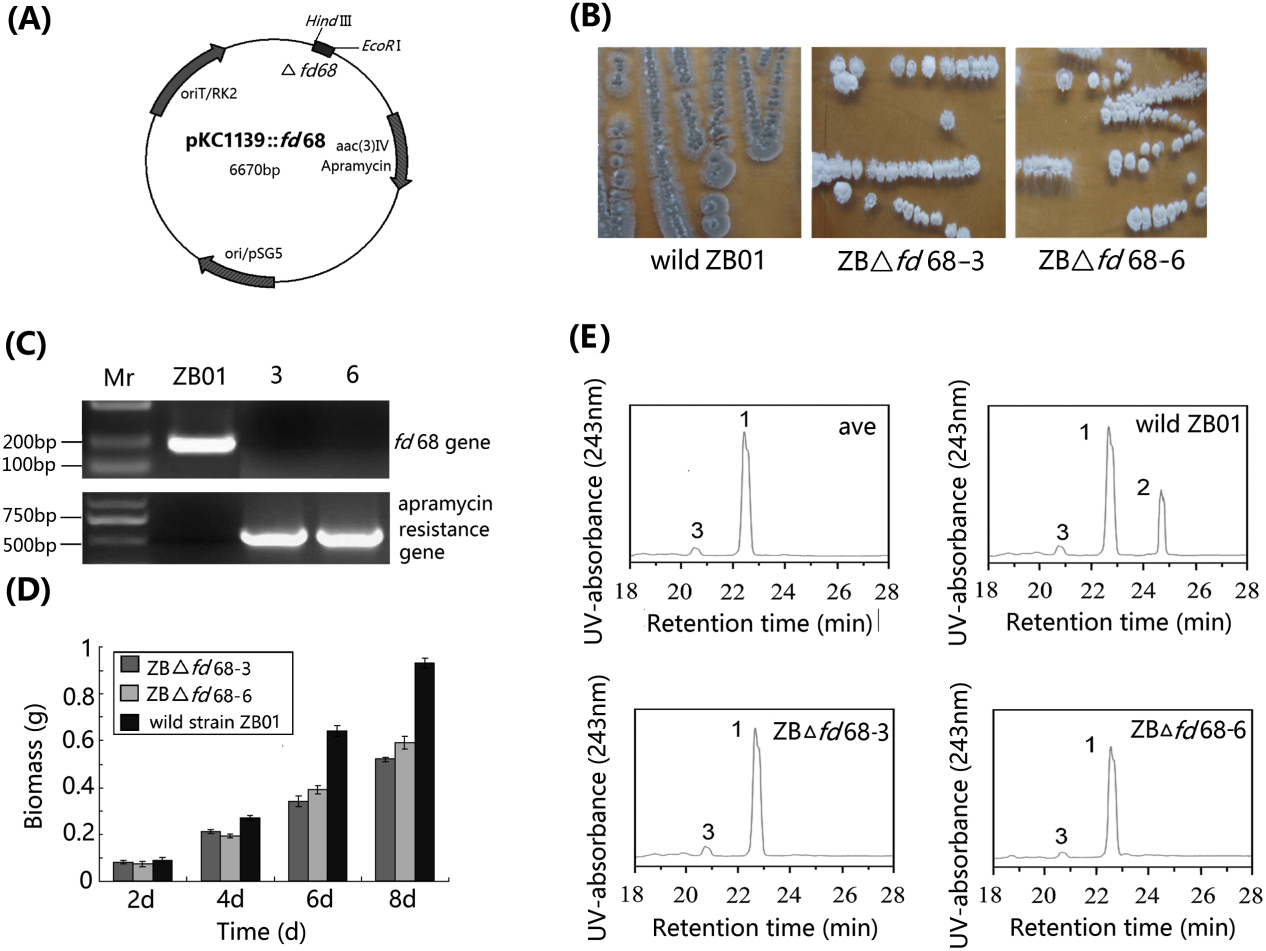
Characterization of *fd*68 gene disruption mutants of *S.ahygroscopicus* ZB01. (A) Map of the fd68 knock-out plasmids pKC1139:: *fd*68. The 172bp *fd*68 fragment named Δfd68 was subcloned into the *EcoR* I and *Hind* III sitesa of lacZα MCS in plasmid pKC1139. (B) Phenotype of wild *S. ahygroscopicus* ZB01 and *fd*68 disruption mutants ZBΔ*fd*68-3 and ZBΔ*fd*68-6 (7 d on YMS medium at 30°C). Note the color changes of the colonies of the strains. (C) PCR analysis of apramycin resistance gene and *fd*68 with primers AF1/AR1 for apramycin resistance gene and fF1/fR1 for *fd*68; Mr, DNA Marker. The line above the lane numbers indicates DNA from wild-type strain *S. ahygroscopicus* ZB01, mutant ZBΔ*fd*68-3 and ZBΔ*fd*68-6. (D) Mycelium dry weights of *fd*68 disruption mutants ZBΔ*fd*68-3, and ZBΔ*fd*68-6 and wild-type *S. ahygroscopicu* ZB01 at different incubation times in YEME. 10^8^ spores of strains were inoculated in 250 ml flasks with 80 ml liquid YEME medium and cultured for 8 d, the mycelium were collected and dried at 70°C for 1 d. Error bars represent the standard deviation of three replicas in three independent experiments. (E) HPLC analysis of the products of avermectin catalyzed by avermectin standard, wild *S. ahygroscopicus* ZB01, ZB△*fd*68-3 and ZB△*fd*68-6. The peaks of avermectin B1a and metabolites are indicated. The 1 represents the peak of avermectin B1a, 2 represents the peak of 4″-oxo-avermectin, and 3 represents the peak of avermectin B1b. The retention times for avermectin B1a is 22.5 min, for 4″-oxo-avermectin B1a is 24.7 min, and for avermectin B1b is 20.7 min.

To ellucidate the function of *fd*68 gene, a *fd*68 gene disruption vector pKC1139:: *fd*68 was constructed (Fig. 3A) and transformed into ZB01. Two stable G418 resistant transformants ZB△fd68-3 and ZB△fd68-6 were selected. The plasmid. pKC1139:: *fd*68 could not be extracted from these two mutants (data not shown), so *fd*68 and apramycin resistance genes were analyzed by PCR using the genomic DNA of the mutants as templates. There was an intact *fd*68 gene (about 200 bp) and no apramycin resistance gene in wild *S. ahygroscopicus* ZB01, while no intact *fd*68 gene was amplified at the presence of apramycin gene fragments (about 500 bp) in ZB△fd68-3 and ZB△fd68-6 (Fig. 3B), suggesting that pKC1139::*fd*68 had been integrated into the chromosome of ZB01 and disruption had occurred in ZB△fd*68-*3 and ZB△fd*68-*6.

The colony morphologies of wild *S. ahygroscopicus* ZB01, ZB△*fd*68-3 and ZB△*fd*68-6 on YMS agar were similar during the first four days, but the ZB01 colonies started to turn gray gradually from the fifth day and most colonies were gray till 7 days, while the ZB△*fd*68-3 and ZB△*fd*68-6 colonies were still white at the seventh days (Fig. 3C). ZB01 and the two mutant strains produced similar amounts of spores as observed under an optical microscope, but the two mutant strains showed a 36–48% decrease in mean biomasses from the forth to the six day. (Fig. 3D).

HPLC analysis of the metabolites of avermectin was presented in Fig. 3E. The *S. ahygroscopicus* ZB01 could regiospecifically oxidize avermectin to 4″-oxo-avermectin, as exhibited by a peak at 24.5 min corresponding to 4″-oxo-avermectin, whereas the *fd*68 disrupted mutant strains ZB△*fd*68-3 and ZB△*fd*68-6 were unable to oxidize the substrate, demonstrating that *fd*68 is a key electron transfer protein in oxidation of avermectin by CYP107Z13 in ZB01.

### Characterization of FdR18 and FdR28

**Figure 4:**
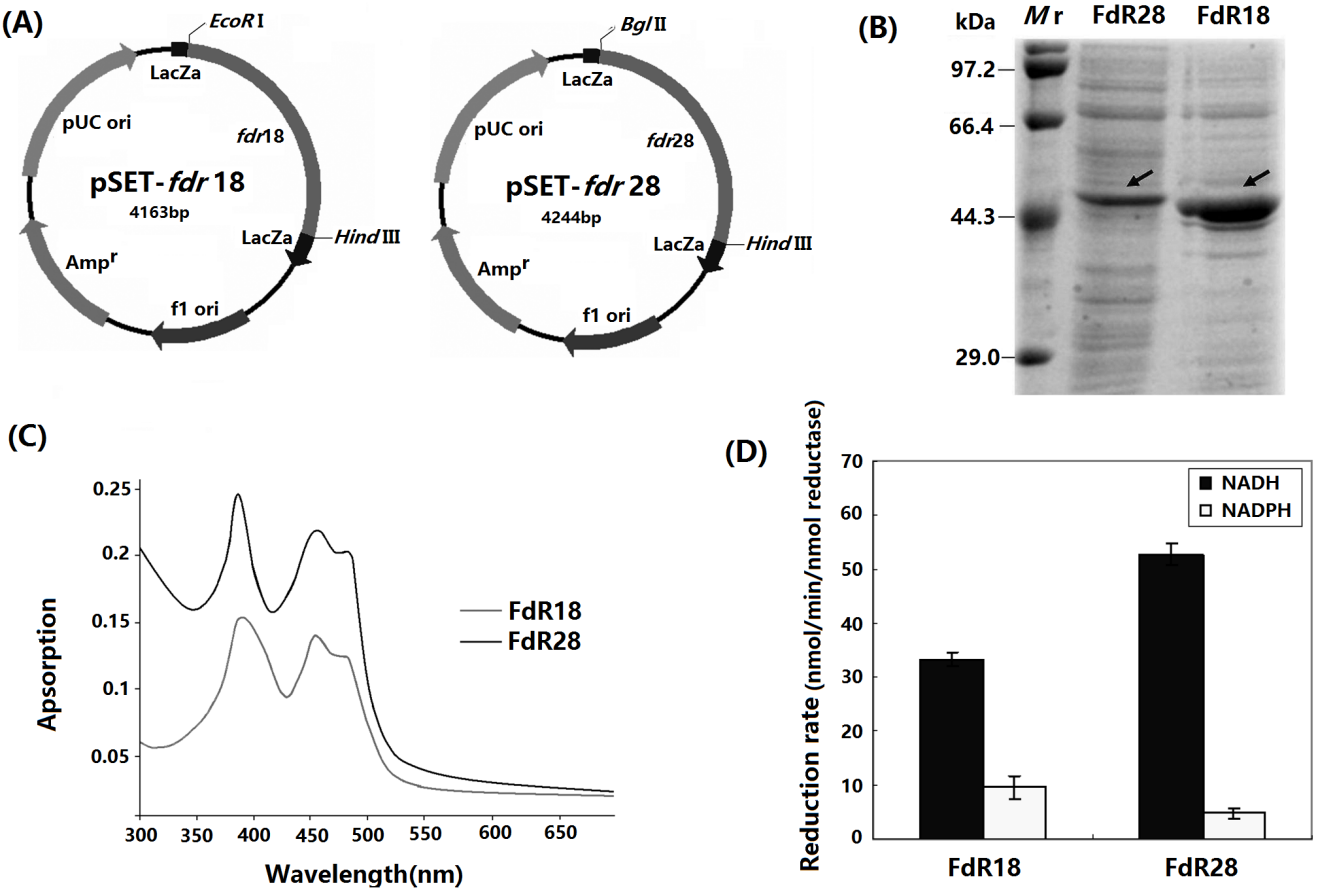
Expression and characterization of FdR18 and FdR28. (A) Recombinant expression vectors pRSET-*fdr*18 and pRSET-*fdr*28. (B) SDS-PAGE analysis of recombinant proteins FdR18 and FdR28 expressed by *E. coli* BL21 (DE3). Mr: protein markers. (C) UV-visible spectra of purified FdR18 and FdR28. Spectra were recorded at ambient temperature in 50 mM Tris buffer (pH 7.5). (D) DCPIP reduction activities of purified FdR18 and FdR28, measured in the presence of 200 uM NADH (▪) or NADPH (□).

pRSET-*fdr*18 and pRSET-*fdr*28 (Fig. 4A) were constructed and transformed into *E. coli* BL21(DE3), the resultant transformants were named *E. coli*-*fdr*18 and *E. coli*-*fd*r28 respectively. The recombinant FdR18 and FdR28 proteins were then expressed and purified. The molecular weight of FdR28 was greater than FdR18 on SDS-PAGE (Fig. 4B). UV-visible spectra analysis demonstrated that absorption peaks appeared at 388, 453, and 482 nm for oxidized FdR18 and at 386, 455, and 486 nm for FdR28 (Fig. 4C). The electron transport rates of FdR18 and FdR28 for NADH and NADPH were detected using DCPIP as the electron acceptor. The *K*_m_ and K_cat_ of FdR18 for NADH, evaluated using DCPIP, was 64 µM and 121 min^−1^, whereas those of FdR28 for NADH were 25.4 µM and 386 min^–1^, respectively. Both FdR18 and FdR28 proteins showed higher electron transport activity against NADH than NADPH, showing that both of the proteins are possible NADH-dependent FdRs (Fig. 4D).

### Whole-cell Biocatalytic Systems for Oxidation of Avermectin Using *E. Coli*

**Figure 5:**
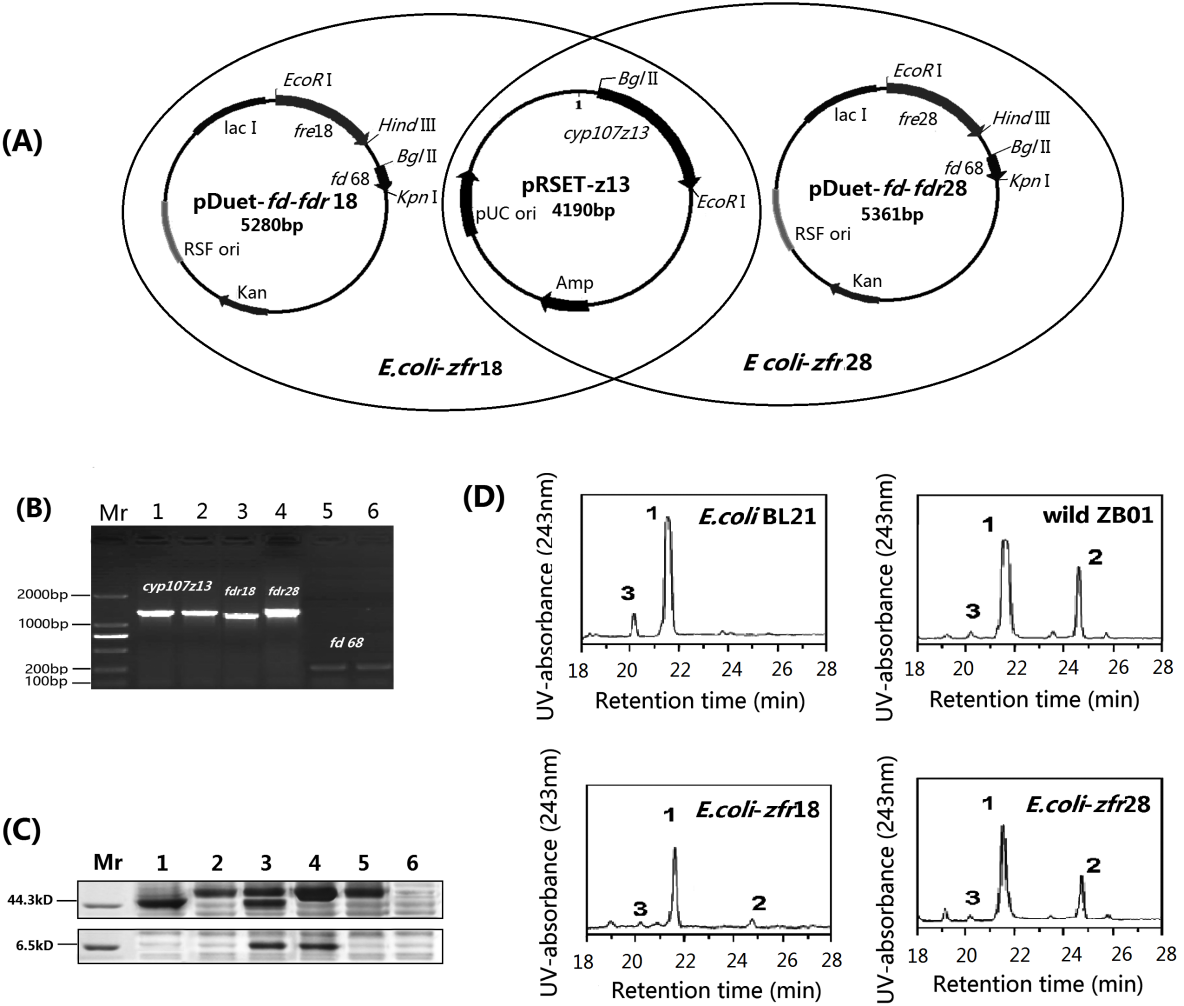
Construction and characterization of whole-cell catalytic system for oxidation of avermectin. (A) Construction of *cyp107z13* gene expression vector pRET-z13, co-expression vector pDuet-*fd*-*fdr*18 and pDuet-*fd*-*fdr*28. *E. coli-zfr*18 was *E. coli* BL21 (DE3) containing pRSET-z13 and pDuet-*fd*-*fdr*18, *E. coli-zfr*28 was *E. coli* BL21 (DE3) containing pRSET-z13 and pDuet-*fd*-*fdr*28. (B) PCR analysis of *cyp107z13*, *fd*68, *fdr*18 and/or *fdr* 28 genes in *E. coli-zfr*18 and *E. coli-zfr*28. *cyp107z13* with primers: z13F+z13R, *fd*68 with primers: RfdF+RfdR, 1,3 and 5: *E. coli-zfr*18; 2,4 and 6: *E. coli-zfr*28. *fdr*18 with primers: Rzre1F+Rzre1R, *fdr*28 with primers: Rzre1F+Rzre2R. PCR products of *cyp107z13*, *fd*68, *fdr*18 and *fdr*28 are 1920 bp, 195 bp, 1263 bp and 1344 bp respectively. (C) SDS-PAGE analysis of recombinant proteins expressed by *E. coli-zfr*18 and *E. coli-zfr*28. Mr: protein markers; 1: *E. coli-fdr*18; 2: *E. coli-fdr*28; 3: *E. coli-zfr*18; 4: *E. coli-zfr*28; 5: *E. coli-z13*; 6: *E. coli* BL21 (DE3). (D) HPLC analysis of the products of avermectin catalyzed by *E. coli* BL21(DE3), wild *S. ahygroscopicus* ZB01, *E. coli-zfr*18 and *E. coli-zfr*28. The peaks of avermectin B1a and metabolites are indicated. The 1 represents the peak of avermectin B1a, 2 represents the peak of 4″-oxo-avermectin, and 3 represents the peak of avermectin B1b. The retention times for avermectin B1a is 21.6 min, for 4″-oxo-avermectin B1a is 24.8 min, and for avermectin B1b is 20.3 min.

For co-expressing CYP107z13, Fd68 and FdR18/FdR28 in *E. coli*. pRSET-*z13*, pDuet-*fd*-*fdr*18 and pDuet-*fd*-*fdr*18 were constructed (Fig. 5A). pRSET-z13 and pDuet-*fd*-*fdr*18 were co-transformed into *E. coli* BL21 (DE3) and the resultant transformant *E. coli*-*zfr*18, pRSET-*z13* and pDuet-*fd*-*fdr*18 were co-transformed into *E. coli* BL21 (DE3) and transformant *E. coli*-*zfr*28 were obtained. Both of *E. coli*-*zfr*28 and *E. coli*-*zfr*28 showed *cyp107z13*, *fd*68 and *fdr*18/28 genes by PCR amplifying (Fig. 5B). The expressed target proteins from *E. coli*-*zfr*18 and *E. coli*-*zfr*28 were analyzed by SDS-PAGE (Fig. 5C).

HPLC was performed to evaluate whole-cell regiospecific oxidase activities of *E. coli*-*zfr*18 and *E. coli*-*zfr*28 for production of 4″-oxo-avermectin. Wild-type ZB01 was employed as positive controls, and *E. coli* BL21(DE3) as negative controls. HPLC analysis of the metabolites of avermectin was presented in Fig. 5D. Wild-type ZB01, *E. coli*-*zfr*18 and *E. coli*-*zfr*28 can all regiospecifically oxidize avermectin to 4″-oxo-avermectin (Fig. 5D). Conversion efficiency was found to be 16% in wild type ZB01 and zero in negative control strains, while it was 11.2% and 0.6% in *E. coli*-*zfr*28 and *E. coli*-*zfr*18 respectively. These results showed that both FdR18 and FdR28 sustained the oxidizion activity of avermectin to form 4″-O-avermectin, with electron transfer efficiency of FdR28 to Fd68 higher than that of FdR18 to Fd68.

## Discussion

The electron transferation of P450s from *Streptomyces* are of the classical class I system, which constitutes ferredoxin reductase (FdR), ferredoxin (Fd), and P450. Electrons are delivered from reduced pyridine nucleotide coenzymes NAD(P)H to P450 via an FdR with flavin adenine dinucleotide and an iron–sulfur protein Fd [26]. There are many CYP450 genes and relatively few Fd and FdR genes in *Streptomyces*. Ninteen CYP450s, two Fd genes and four FdR genes were found in *S. Peuculate* genome [27], eighteen CYP450s, six Fd and four FdR genes were found in *S. coelicolor* A3(2) genome, and thirty-three CYP450s, nine Fd and six FdR genes were found in *S. avermitilis* genome [28]. CYP450 of *Streptomyces* has a high specificity to Fd and FdR as the electron transfer proteins for special catalytic function. CYP105A1 can only accept electrons transported from Fd1 and CYP105B1 can only accept electrons from Fd2 to perform the 7-ethoxycoumarin hydroxylation reaction in *S. griseolus* [10]. The electron transport pathway of hydroxylation fatty acid CYP105D5 in *S. coelicolor* A3(2) was NADH→FdR1→Fd4→CYP105D5 [26]. In our previous studies, we found that *S. ahygroscopicus* ZB01 had a strong catalytic activity for the region-specific oxidation of 4″-OH of avermectin to form 4″-oxo-avermectin. Resting *S. ahygroscopicus* ZB01 cells can convert 36% of the avermectin substrate to 4″-oxo-avermectin in 72 h at avermectin concentrations of 1 g l^−1^ measured by HPLC [17], whereas the resting *S. tubercidicus* I-1529 cells can convert 16% of the avermectin substrate to 4″-oxo-avermectin in 96 h at avermectin concentrations of 0.75 g l^−1^ which was reported to be the highest biocatalytic activity reported by Molnár [5]. CYP107Z13 was responsible for this regio-specific oxidation of avermectin. In this study, we cloned one Fd gene *fd*68, two FdR genes *fdr*18 and *fdr*28, and found that there exist the electron transfer pathway NADH→FdR18/FdR28→Fd68→CYP107Z13 in ZB01 in oxidation of avermectin to form 4″-oxo-avermectin.

Many studies on *Streptomyces* P450s have been reported. However, only a few studies were focused on their electron-transport proteins Fds and FdRs [29]–[31]. [2Fe-2S] Fd from *Pseudomonase putida* (CamB) plays a role in electron transfer from FdR from *P. putida* (CamA) to CYP107P3 of *S. griseus*, and mediate the O-dealkylation of 7-etgoxycumarin [32]. CamB can also transfer electron to CamA and sustain the hydroxylation of daidzein by CYP107H1 from *Bacillus subtilis* [15]. Fd containing [3Fe-4S] cluster from *S. clavuligerus* was shown to be involved in clavulanic acid biosynthesis. *Mycobacterium tuberculosis* [3Fe-4S] Fd can pass electron to CYP51, a 14α-sterol demethylase [26]. Thirteen CYP107Z genes had been found till now in *Streptomyces,* all of which exhibited regioselective oxidation activities to avermectin. Only Fd232 in *S. tubercidicus* I-1529 and Fd233 in *S. tubercidicus* R-922 were identified to be the electron transfer proteins of CYP107Zs for oxidation of avermectin. Both Fd233 and Fd232 are [3Fe-4S] Fds and have a high homogeneity (only one amino acid difference) and their flanking sequences are also of high homogeneity [5]. We speculated that Fd, which can transfer electrons to CYP107Z13 for oxidation of avermectin in ZB01, might be homologous to Fd232 and Fd233. Thus we designed primes F1/R1 according to the homologous flanking sequences of *fd*232 and *fd*233 and successfully cloned *fd*68 gene from ZB01 by PCR. We expressed *fd*68 in a prokaryotic expression system and purified a 7.1 kDa recombinant protein Fd68. The expression amount and the activity of purified Fd68 were relatively low (data not shown). This may partially due to the instability, small molecular weight and low expression level of Fd68 by *E. coli.*

It was generally believed that the homologous double exchange mutants were more stable than single exchange mutants. We constructed *fd*68 homologous double exchange (data not shown) and single exchange gene disruption plasmids respectively by utilizing pKC1139, and only homologous single exchange mutants were obtained. Both the two *fd*68 gene disruption mutants ZB△*fd*68-3 and ZB△*fd*68-6 were genetically stable and lost the activity of oxidation avermectin, showing that Fd68 is a key electron transfer protein of oxidising avermectin by CYP107Z13 in ZB01.

Both of the oxidized FdR18 and FdR28 showed typical UV-visible absorption spectrum of FAD dependent enzyme [33], which are similar to that of PdR in *Pseudomonas putida* [34], ArR of *Novosphingobium aromaticivorans* [11], ApbE in *Salmonella enterica* and FdRs in *S. coelicolor* A3(2) [35] and *S. Griseus* [9]. Both FdR18 and FdR28 are possibly NADH-dependent FdRs, which is similar to FdRs in *S. coelicolor A3(2)* and *M. tuberculosis* [26], [36]. Both *fdr*18 and *fdr*28 knockout mutants in this study can oxidize avermectin to form 4″-O-avermectin, although both the conversion efficiency decreased about 46–60% comparing to that of wild ZB01 (data not shown). We had not got the *fdr*18-*fdr*28 double gene-disruption mutants. To determine whether FdR18 and FdR28 are electron transfer proteins in the catalytic reaction of oxidizing avermectin by CYP107Z13, we constructed two whole-cell biocatalytic systems co-expressing CYP107Z13, Fd 68 and FdR18/FdR28 in *E. coli* BL21 (DE3), using two compatible vectors pRSFDuet-1 and pRSF-1, and clarified that both of FdR18 and FdR28 could sustain the electron transfer activities to oxidise avermectin by CYP107Z13 [37], [38].

FdR and Fd coding genes in *Streptomyces* may clustered with P450 genes, some of which are distributed freely in the genome. In the *S. coelicolor* A3(2) genome, *fdr*1, *fd*4 with *cyp105d5* and *fdr*2, *fd*1 with *cyp158a1* are located close together, but *fdr*3, *fdr*4, and the other four *fd*s are located far from each other with other P450 genes [26]. Six *fdr*s and nine *fds* are present in *S. avermitilis* genome, only *fdr*B and *fdx*B with *cyp105q1* are located close together [28]. *fd*68, *fdr*18 and *fdr*28 were not clustered with *cyp107z13*. However, there is another unknown P450 gene at 49 bp upstream of *fd*68 (data not shown), which hints that Fd68, FdR18 and FdR28 may not be the natural electron transport proteins for CYP107z13 in ZB01.
